# Functional analysis of conserved aromatic amino acids in the discoidin domain of *Paenibacillus *β-1,3-glucanase

**DOI:** 10.1186/1475-2859-8-62

**Published:** 2009-11-25

**Authors:** Yueh-Mei Cheng, Feng-Chia Hsieh, Menghsiao Meng

**Affiliations:** 1 Graduate Institute of Biotechnology, National Chung Hsing University, 250 Kuo-Kuang Rd, Taichung, 40227, Taiwan; 2 Biopesticides Division, Taiwan Agricultural Chemicals and Toxic Substances Research Institute, Council of Agriculture, 11 Kuang Ming Rd, Wufeng, Taichung Hsien, 413, Taiwan

## Abstract

The 190-kDa *Paenibacillus *β-1,3-glucanase (LamA) contains a catalytic module of the glycoside hydrolase family 16 (GH16) and several auxiliary domains. Of these, a discoidin domain (DS domain), present in both eukaryotic and prokaryotic proteins with a wide variety of functions, exists at the carboxyl-terminus. To better understand the bacterial DS domain in terms of its structure and function, this domain alone was expressed in *Escherichia coli *and characterized. The results indicate that the DS domain binds various polysaccharides and enhances the biological activity of the GH16 module on composite substrates. We also investigated the importance of several conserved aromatic residues in the domain's stability and substrate-binding affinity. Both were affected by mutations of these residues; however, the effect on protein stability was more notable. In particular, the forces contributed by a sandwiched triad (W1688, R1756, and W1729) were critical for the presumable β-sandwich fold.

## Background

The discoidin domain (DS domain) is a structural and functional motif that is appended, singly or in tandem, to various eukaryotic and prokaryotic proteins [1]. The first DS domain was identified in the amoeba *Dictyostelium discoideum *and described as a lectin with high affinity for galactose and galactose derivatives [2]. It should be noted that the domain is also referred to as F5/8C due to its presence at the carboxyl-terminus of blood coagulation factors V and VIII. The DS domain binds a wide variety of ligand molecules, including phospholipids, carbohydrates, and partner proteins, thus enabling its cognate protein to participate in various physiological functions such as cellular adhesion [3,4], migration [5], neural development [6,7], and nutrition assimilation [8,9]. A subgroup of the domain possessing carbohydrate-binding ability is also classified as the carbohydrate-binding module family 32 (CBM32) [10]. Due to the recent progress of genome projects, the number of CBM32 members has increased significantly over a short period time. However, most of these members have not been functionally characterized.

The structure of several DS domains has been determined and deposited in the PDB [11]. The DS domain comprises approximately 150 amino acid residues, arranged into a β-sandwich fold with several flexible loops. Presumably, the β-sandwich fold is stabilized predominantly by hydrophobic interactions. The variability within the loops has been suggested to account for the diverse binding spectrum of the DS domain [12]. Co-crystallizations of CBM32 members and their ligands, such as the module of *Clostridium perfringens *N-acetylglucosaminidase with β-galactosyl-1,4-β-N-acetylglucosamine or the module of *Micromonospora viridifaciens sialidase *with lactose, demonstrate that the protruding loops form the ligand binding site [13,14].

Recently, a β-1,3-glucanase consisting of 1793 amino acid residues [GenBank: DQ987544] was isolated from *Paenibacillus sp*. BCRC 17245 and was characterized [15]. This β-1,3-glucanase (referred to as LamA hereafter) is highly modular, containing a signal sequence, three repeats of the S-layer homologous module, a segment with unknown function, a catalytic module of glycoside hydrolase family 16 (GH16), four repeats of CBM4 family, and a F5/8C module from amino- to carboxyl-terminus. Differential properties between two truncated proteins (GH16 and GH16 tagged with the F5/8C module) suggested that the carboxyl-terminal F5/8C has an ability to complex with polysaccharides containing β-1,3-, β-1,3-1,4-, and β-1,4-glucosidic linkages and such ability conferred greater antifungal activities to GH16 on the growth of *Candida albicans *and *Rhizoctonia solani*. In addition, the presence of the F5/8C module enhances the hydrolyzing activity of the catalytic module to various polysaccharides. To better understand the F5/8C module in terms of its structure and function, the module alone was expressed in *E. coli *and characterized biochemically in this study. In addition, functions of several conserved aromatic amino acid residues in the module were investigated by mutagenesis.

## Materials and methods

### Chemicals

Laminarin, chitin (from crab shells), and lichenan were purchased from Sigma, while Avicel PH101 was purchased from Fluka. The chitin was treated with phosphoric acid prior to use [16].

### Plasmids

pET-C and pET-CF were used for expression of the truncated proteins GH16 and the GH16 fused with F5/8C, respectively [15]. To express the F5/8C module, the pET-F plasmid was generated by PCR-based deletion mutagenesis (QuickChange Site-Directed Mutagenesis Kit, Stratagene) using pET-CF as the template. The PCR was conducted for 35 cycles (95°C, 30 s; 60°C, 30 s; 72°C, 6 min) followed by a 10 min extension at 72°C in a 50 μl solution that contained 10 ng pET-CF, 0.32 mM each of the 5'-phosphorylated primers (5'-TATGCAGGGAATACGGTCTCC and 5'-CGAATTCGGATCCTGGCTGTG), 0.2 mM of each of the dNTPs, and 2.5 U of *Pfu *polymerase. The PCR product was recovered and self-ligated to become pET-F. A similar PCR protocol, with specific divergent primers, was used to create the specific mutation of pET-F for the production of the mutant F5/8C module. The desired mutations were confirmed with ABI Prism 3773 auto sequencer.

### Protein preparation

*E. coli *BL21(DE3) strain, harboring the desired derivatives of pET-F, was grown at 30°C in 500 mL of 2× TY medium (16 g/L tryptone, 10 g/L yeast extract, and 5 g/L NaCl) supplemented with ampicillin 100 μg/mL until the OD_600 _reached around 1. Isopropyl β-D-1-thiogalactopyranoside (final concentration of 1 mM) was added to the medium and the culture was continued for 7-8 h. The cells were harvested by centrifugation, suspended in 10 mL lysis buffer (20 mM Tris-HCl [pH8.0], 500 mM NaCl, 10% glycerol, 5 mM phenylmethylsulfonyl fluoride, and 5 mM β-mercaptoethanol), and disrupted by ultrasonic treatment. The clarified protein solution was mixed with Ni-NTA resin (Promega) in the presence of 10 mM imidazole. The mixed resin was washed extensively with wash buffer (10 mM phosphate [pH 7.0], 500 mM NaCl, 10% glycerol, and 40 mM imidazole), and the bound protein was eluted with wash buffer containing 500 mM imidazole. The eluted protein was then passed through a Sephacryl S-300 gel filtration column (Hiprep 16/60, Pharmacia) equilibrated with 10 mM sodium acetate [pH 5.8] and 10% glycerol for further analysis and molecular weight estimation. Protein concentration was determined by the Coomassie blue method using bovine serum albumin (BSA) as the standard.

### Instrumental analysis

The sedimentation velocity study was carried out with an analytical ultracentrifuge (Beckman Coulter XL-A) at 20°C and 42000 rpm. The concentration of the protein sample was adjusted to an OD_280 _of 0.5 (0.17 mg/mL) in 10 mM phosphate buffer [pH 7.0] and 10% glycerol. The UV absorption at 280 nm was scanned 200 times in continuous mode and the data were analyzed with Sedfit94 software to obtain the differential concentration distribution *c*(*s*).

The circular dichroism (CD) spectrum within the range of 190 to 360 nm was recorded at room temperature using a Jasco J-815 CD spectrometer fitted with a quartz cell of 5 mm path length. The concentration of the protein sample was adjusted to 0.2 mg/mL in a 10 mM phosphate buffer [pH 7.0]. The data were collected using JWSTDA32 software and analyzed with a CONTINLL program in CDPro software. The protein set SP37 was used as the reference during analysis.

Differential scanning calorimetry (DSC) was performed using a calorimeter (N-DSCIII, TA). Measurements were made with a scan rate of 2°C/min over a 20-80°C range, using a sample concentration of 1 mg/mL in the 10 mM phosphate buffer [pH 7.0]. The data were analyzed with CpCalc OLE 2.0 software.

### Substrate binding assays

The affinity of the protein to soluble polysaccharides was assayed by affinity electrophoresis [17] in which 0.3% (w/v) laminarin was included in a native 12% polyacrylamide gel (PAGE). Electrophoresis was performed at 80 V at 4°C.

The affinity of the protein for insoluble polysaccharides was determined by a pull-down assay; the protein (20 μg/mL) and substrates at indicated concentrations were mixed in a final volume of 300 μL in a 50 mM sodium acetate buffer [pH 5.8]. After 1 h of shaking at 37°C, the supernatant was recovered by centrifugation at 16,000 × *g *for 5 min. The pellet was washed once and suspended in 300 μL of the same buffer. The fraction of protein in the supernatant and the pellet was estimated by 12% sodium dodecyl sulfate (SDS)-PAGE and Coomassie blue staining. The apparent binding affinity (*K*d) of the protein for chitin was calculated using GraFit5 software according to the dependence of the protein fraction bound to chitin on the amounts of chitin used. *K*d was defined as the amount of chitin required for reaching 1/2 of the maximal binding of the protein.

### Antifungal activity assays

Macroconidia of *Fusarium oxysporum *f. sp. *lycopersici *and conidia of *Glomerella cingulata *were washed out from the respective hypha with a 10 mM sodium acetate buffer [pH 5.8] containing 10% glycerol and adjusted to a concentration of 2 × 10^5^/mL. To determine the protein's effect on conidial germination, equal volumes of the conidia solution and protein sample (12.5 μM) were mixed and incubated at 22°C under moisture condition for 16 h. Growth of hypha from the conidia was observed under a light microscope and the hyphal length was measured using Leica Image-ProPlus 4.5 software.

## Results

### Protein expression and purification

**Figure 1:**
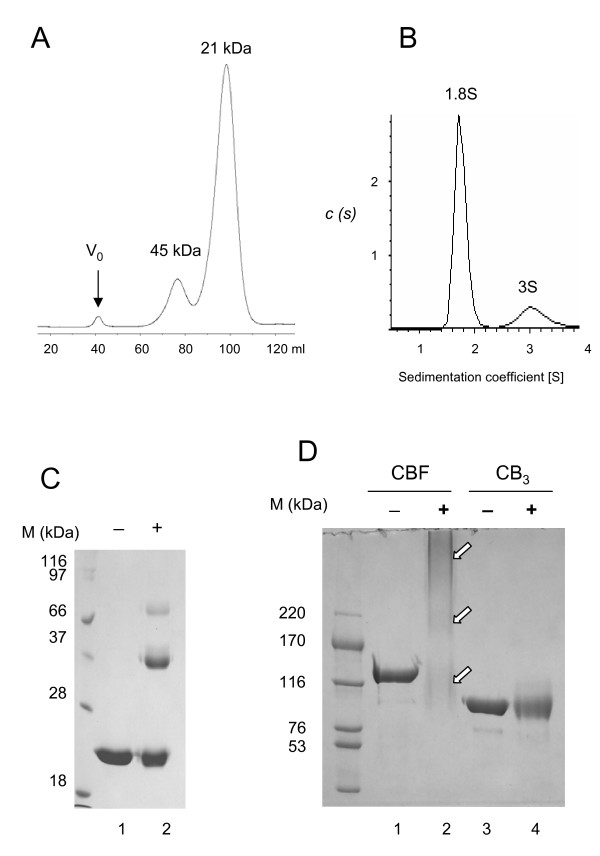
**Monomer to dimer ratio of the F5/8C module of LamA**. (A) Elution profile of the purified protein using a Sephacryl S-300 column. The V_o _indicates the void volume. The estimated molecular masses of the eluted peaks are indicated. (B) Sedimentation velocity studies. The condition for analytical ultracentrifugation and data analysis are described in the Materials and Methods. (C) Glutaraldehyde cross-linking of the purified F5/8C module. The protein (0.65 mg/mL) was treated with 0.05% glutaraldehyde at room temperature for 1 h and resolved on a 12% SDS-PAGE gel. Lanes 1 and 2 contain the protein sample without and with the treatment of glutaraldehyde, respectively. (D) Glutaraldehyde cross-linking of two larger truncated proteins of LamA (CBF and CB_3_). The proteins (0.3 mg/mL) were treated with 0.05% glutaraldehyde at room temperature for 40 min and resolved on an 8% SDS-PAGE gel. The arrows point to the putative migration zones of monomeric, dimeric, and multimeric CBF after glutaraldehyde treatment.

To have a better understanding of the function of the carboxyl-terminal F5/8C module of LamA, pET-F was constructed to produce a recombinant polypeptide consisting of an amino-terminal hexahistidine tag and amino acid residues 1636-1793 of LamA in *E. coli*. The recombinant F5/8C was purified by immobilized metal affinity and gel filtration chromatography. The gel filtration profile suggested that a portion of the recombinant protein exists as a dimer (Fig. 1A). This observation was verified by the observation of two peaks with different sedimentation coefficients by centrifugal analysis (Fig. 1B). The dimer fraction was estimated to be approximately 15% according to both analyses. The existence of a dimer was then confirmed by treating the recombinant F5/8C with 0.05% glutaraldehyde followed by SDS-PAGE (Fig. 1C). A significant fraction of the F5/8C migrated with the apparent molecular mass of a dimer by SDS-PAGE after being cross-linked with glutaraldehyde (Fig. 1C, lane 2). To know whether the dimerization also occurs in larger truncated fragments of LamA, two recombinant proteins, CB_3 _and CBF, were treated with glutaraldehyde. The 92-kDa CB_3 _contains GH16 followed by three repeats of CBM4, while the 144-kDa CBF contains GH16 followed by the polypeptide all the way down to the C-terminal F5/8C module (15). Glutaraldehyde treatment blurred the protein band of CB_3 _on SDS-PAGE gels (Fig. 1D, lane 4). Intramolecular cross-linking among the various modules may account for the smear. As to CBF, protein molecules corresponding to monomers, dimers, and multimers were vaguely shown on SDS-PAGE gels after glutaraldehyde treatment (Fig. 1D, lane 2). The results suggest that the F5/8C module may promote the dimerization of LamA.

### Polysaccharide-binding activity

**Figure 2:**
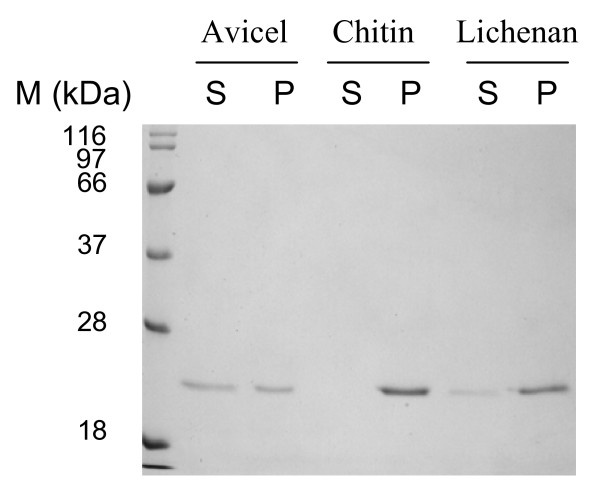
**Binding of the F5/8C module of LamA to insoluble polysaccharides**. The purified protein (20 μg/mL) and the indicate substrate (25 mg/mL) were thoroughly mixed at 37°C for 1 h. The amount of protein remaining in the supernatant (S) and co-precipitating with the substrate (P) were examined by SDS-PAGE.

A pull-down assay was used to determine whether the F5/8C module alone binds insoluble polysaccharides. The purified protein was constantly mixed with various substrates, including Avicel, chitin and lichenan. One hour later, the amounts of F5/8C remaining in the supernatant or co-precipitating with the substrates was examined by SDS-PAGE (Fig. 2). The recombinant F5/8C bound the insoluble substrates and the binding affinity determined was as follows: chitin was the highest, followed by lichenan, then Avicel.

### Antifungal activity

**Figure 3:**
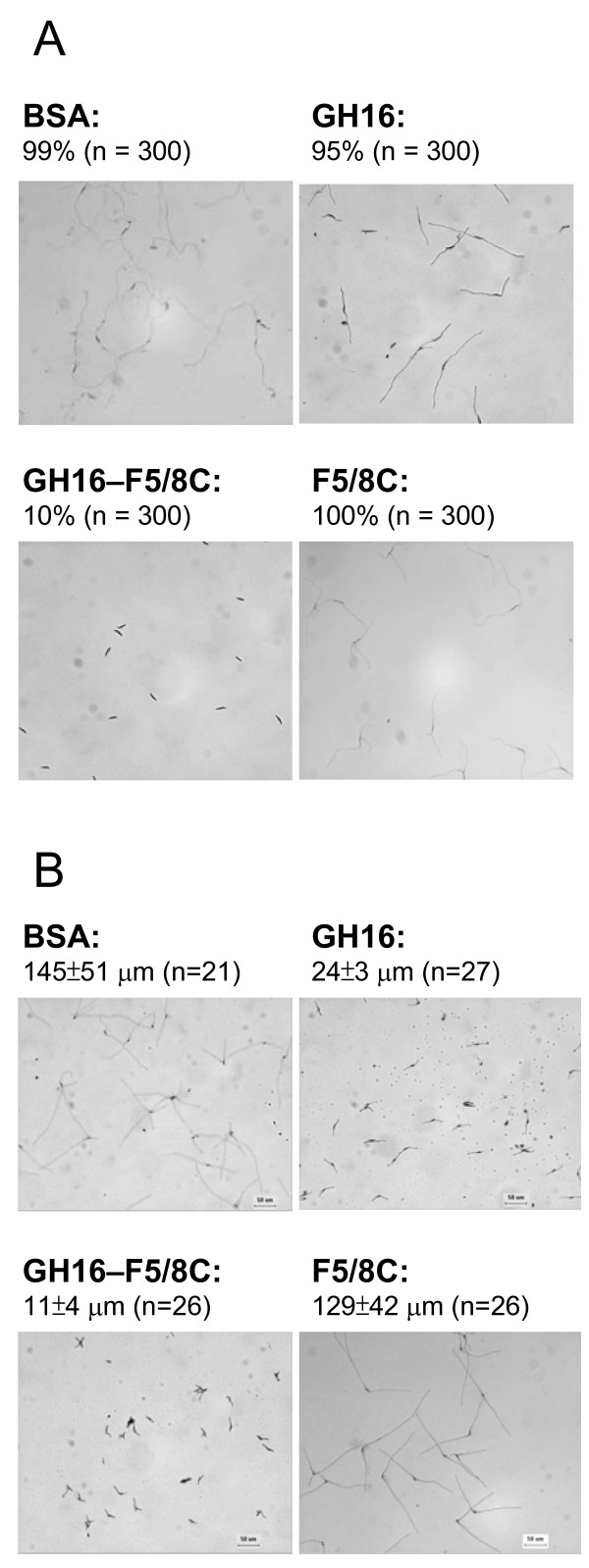
**Effects of the truncated proteins derived from LamA on conidial germination**. Germination of *F. oxysporum *macroconidia (A) and *G. cingulata *conidia (B) in a buffer containing 6.25 μM of either BSA, the GH16 module, the F5/8C module, or the GH16-F5/8C fusion protein. The culture conditions are described in the Materials and Methods. The germination rate (%) and hyphal length (μm) are indicated in panels A and B, respectively.

A previous study showed that appending F5/8C to GH16 could enhance the anti-fungal activity of GH16 on the growth of *C. albicans *and *R. solani*. Therefore, we were interested in knowing whether F5/8C alone is able to interfere with fungal growth. To answer this question, various truncated proteins, including GH16, F5/8C, and GH16-F5/8C, were mixed with macroconidia of *F. oxysporum *and conidia of *G. cingulata*. The germination percentage of *F. oxysporum *macroconidia was not significantly affected by either GH16 or F5/8C, but dropped to 10% with GH16-F5/8C under the assay conditions (Fig. 3A). For *G. cingulata *conidia, none of the treatments prevented germination. However, the lengths of the growing hypha were different in the buffer containing the different proteins (Fig. 3B). GH16 reduced the hyphal length to 16% of the control treated with BSA. The length was further reduced to 8% by GH16-F5/8C. These data suggest that although F5/8C itself does not have an antifungal activity, it does have an auxiliary role in maximizing the inhibitory function of GH16, presumably through rendering GH16 more accessible to the target sites within the fungal cell wall.

### Mutational effects on protein structure

**Figure 4:**
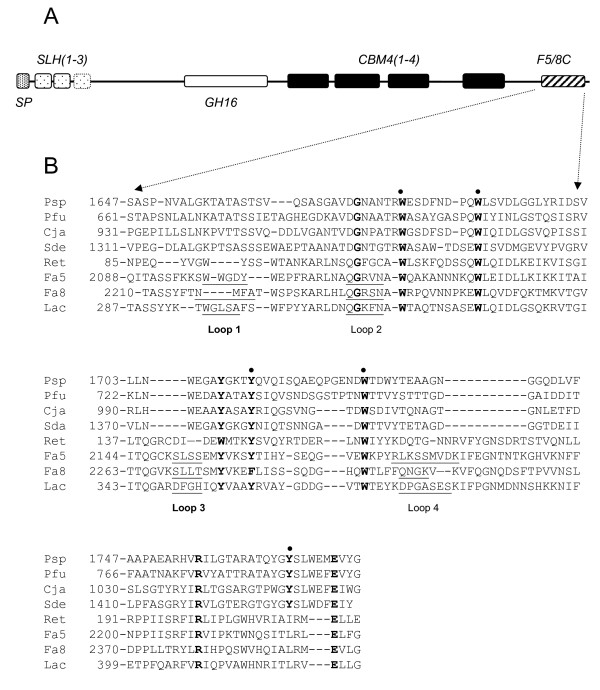
**Amino acid sequence alignment of several DS domains**. (A) Schematic representation of the domain organization of LamA. (B) Sequence alignment, based on the ClustalV method, of the F5/8C module of LamA (Psp; GenBank ABJ15796) with that of *Paenibacillus fukuinensis *chitosanase-glucanase (Pfu; GenBank BAB64835), *Cellvibrio japonicus *cbp32B (Cja; GenBank ACE83872), *Saccharophagus degradans *β-1,3-glucanase (Sde; GenBank ABD82184), retinoschisin (Ret; NP_000321), the C2 domain of human coagulation factor V (Fa5; GenBank AAB59401), the C2 domain of human coagulation factor VIII (Fa8; GenBank AAA52484), and the C2 domain of lactadherin of *Bos taurus *(Lac; NP_788783). Sequence similarities between the DS domain of Psp and Pfu, Cja, Sde, Ret, Fa5, Fa8, and Lac are 39, 36.9, 44.2, 13.0, 13.0, 14.5, and 16.8%, respectively. Conserved residues are shown in bold, while residues mutated in this study are marked with black dots. Sequences that constitute protruding loops in Fa5, Fa8, and Lac are underlined according to PDB code 1CZS, 1D7P, and 3BN6, respectively.  Please note these Proteins can be searched and accessed via http://www.ncbi.nlm.nih.gov/sites/entrez?db=Protein&itool=toolbar

To gain information on the structural properties of the F5/8C of LamA, sequences among several members of the CBM32 family and eukaryotic DS domains were aligned (Fig. 4). Although the overall similarities are low, particularly between prokaryotic and eukaryotic proteins, conservation of several aromatic amino acid residues was noted. Thus, knowing the functions of these residues in the structure and function of the F5/8C is important. Most of the 53 currently classified CBM families employ the side chains of aromatic residues to interact with their ligands. In general, the side chains of tryptophan and tyrosine form the hydrophobic platforms, which can be planar, twisted or form a sandwich, in the carbohydrate-binding sites of CBMs [18]. Therefore, it was intuitional to propose a ligand-binding function to the conserved aromatic residues in the F5/8C of LamA. However, crystal structures of several DS domains indicate that the sugar-binding site relies on the protruding loops, and none of the conserved residues of the F5/8C were predicted to be located in the loop regions (Fig. 4). Therefore, another possible function of the conserved aromatic residues is to maintain the protein structure. To clarify these two possibilities, amino acid residues, including W1679, W1688, Y1714, W1729, and Y1768, of the F5/8C were individually mutated to alanine in this study. Most of the mutant proteins could be expressed properly in *E. coli*, except for W1729A which formed inclusion bodies. Furthermore, the soluble mutant proteins could be purified by the same protocol as the wild type.

**Figure 5:**
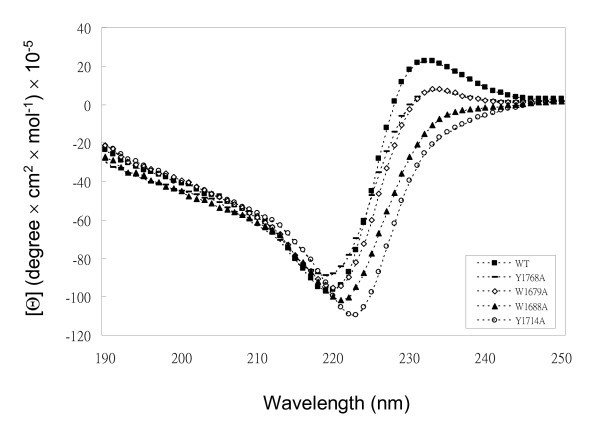
**The circular dichroism spectra of wild-type and mutant F5/8C modules of LamA**. The experimental conditions are described in the Materials and Methods.

Alterations in the secondary structure of the F5/8C by these point mutations were examined by far-UV CD spectra (Fig. 5A). The spectrum of the wild-type F5/8C shows a minimum at 219 nm and a maximum at 232 nm. Secondary structure prediction by the CONTINLL program suggested that there is no α-helix in the structure, consistent with the known β-sandwich fold of DS domains. The mutations caused shifts of the minimum and disappearance of the maximum. The degrees of change in the spectra are in the following order (highest to lowest): Y1714A, W1688A, W1679A, and Y1768A.

**Figure 6:**
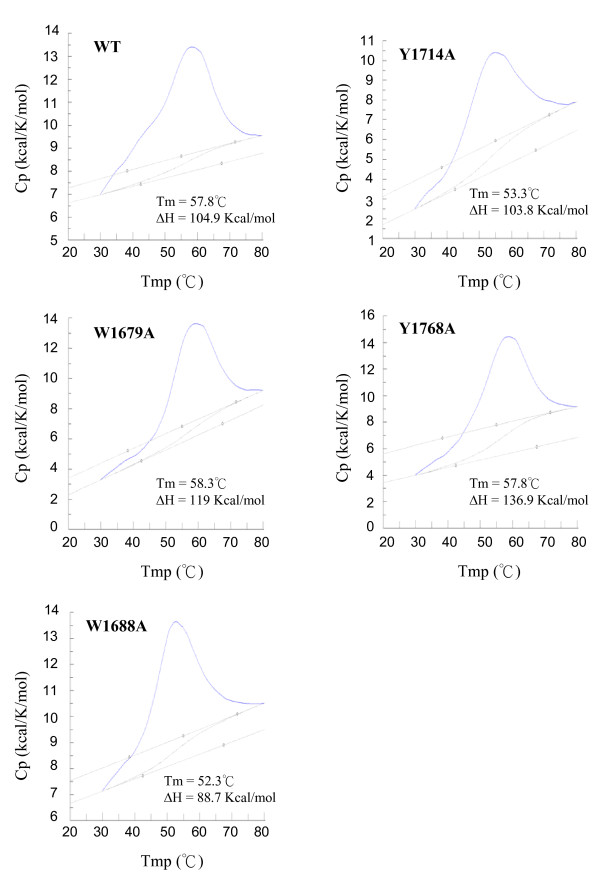
**Differential scanning microcalorimetric plots of wild-type and mutant F5/8C modules of LamA**. The experimental conditions are described in the Materials and Methods.

To determine whether the mutations affected the stability of the F5/8C module, the temperature dependences of the specific heat capacity of the wild-type and mutant modules were analyzed with a differential scanning calorimeter (Fig. 6). The wild-type module exhibited an asymmetric melting curve with a small shoulder preceding the major peak. This melting curve suggests that at least 2 transitions are involved in the protein unfolding process. The major peak had a Tm value of 57.8°C. The replacement of W1688 and Y1714 with alanine decreased the Tm value to 52.3°C and 53.3°C, respectively. In contrast, no significant change in the Tm was observed in response to mutations of W1679A and Y1768A. Of note, the mutations that significantly decreased the Tm also had profound effects on the protein's CD spectra.

### Mutational effects on polysaccharide-binding activity

**Figure 7:**
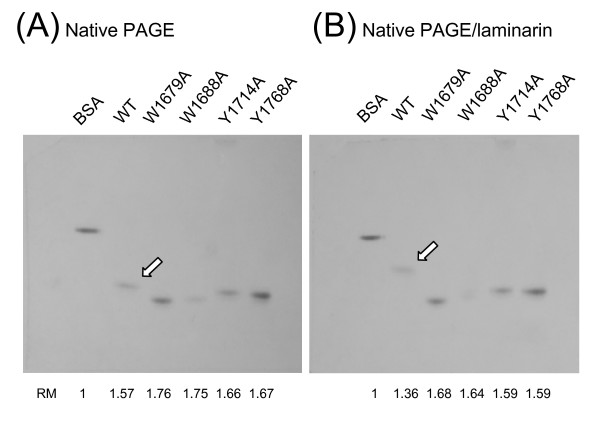
**Affinity electrophoresis of wild-type and mutant F5/8C modules**. The indicated proteins were separated by native 12% PAGE (A) and native PAGE including 0.3% (w/v) laminarin in the separation gel (B). Open arrows point to the migration positions of the wild-type F5/8C module. The relative mobility (RM) of each protein compared with BSA under the given conditions is indicated.

Binding activity of the F5/8C module to laminarin, a soluble β-1,3-glucan, was examined by affinity electrophoresis [17]. In this assay, a stronger binding is manifested by a slower migration of the protein in the gel. Electrophoresis of various F5/8C derivatives in native polyacrylamide gels with or without laminarin is shown in Fig. 7A and 7B, respectively. Two points were noted. First, variant F5/8C derivatives migrated at different rates under the native condition, suggesting that the mutations affected the global protein conformation, consistent with the results of the CD spectra. Second, laminarin retarded the migration of all the F5/8C variants. However, the wild-type proteins displayed greater changes in migration rate compared to the rest of the variants. These data suggest that mutations at W1679, W1688, Y1714, and Y1768 decreased the protein affinity to laminarin.

**Figure 8:**
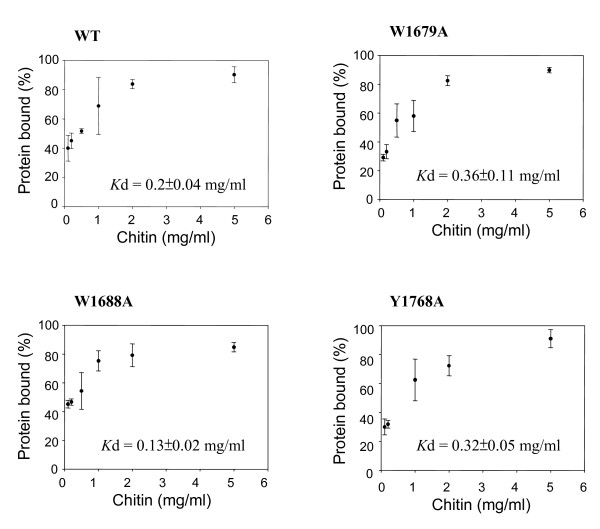
**Dependence of the protein fraction bound to chitin on the chitin concentrations**. Each of the indicated proteins (20 μg/mL) was thoroughly mixed with chitin at the indicated concentrations at 37°C for 1 h. The fractions of the protein that remaining in the supernatant (S) and co-precipitating with chitin (P) were determined by SDS-PAGE. The apparent binding affinity (*K*d) was calculated using GraFit5 software. The data were averages from two independent experiments, each with triplicate samples.

To quantify the mutational effects on polysaccharide binding, we carried out pull-down assays by mixing the F5/8C and chitin at various concentrations. The chitin concentration-dependence of the binding would allow us to estimate the apparent binding affinity (*K*d) of the F5/8C module toward chitin. The results showed that the fraction of WT bound to chitin increased with the increase of chitin concentration and gradually reached a plateau when chitin concentration was more than 2 mg/ml, leading to an approximation of *K*d = 0.2 mg/ml (Fig. 8). The results also indicate a slight increase in affinity for chitin by substituting alanine for W1688. However, the affinity was decreased by the W1679A and Y1768A mutations. The binding affinity of Y1714A could not be correctly estimated because a significant fraction of the mutant protein precipitated during the period of mixing, even in the chitin-absent buffer.

## Discussion

According to the sequence information, a variety of bacterial glycoside hydrolases have been suggested to have one or more DS modules. These hydrolases include, but are not limited to, α-1,3-glucanase [19], β-1,3-glucanase [15,20], β-galactosidase [21], cellulase [22], chitosanase [23], α-1,2-mannanase [24], hyaluronidase [25-27], sialidase [28,29], alginate lyase [30]. Structural and biochemical studies on the modules of *M. viridifaciens *sialidase [14], *Y. enterocolitica *polygalacturonic acid-binding protein [31], and *C. perfringens *N-acetylglucosaminidase [32] have indicated that galactose or its derivatives are the basic units for recognition by family 32 CBMs. In this study, the F5/8C module of LamA was expressed alone in *E. coli *and was characterized in vitro. The affinity electrophoresis results and the pull-down assays indicated that the F5/8C module can bind to laminarin as well as many other insoluble polysaccharides, including chitin, lichenan, and cellulose, suggesting a wide substrate-binding spectrum of family 32 CBMs.

With the broad binding specificity, the carboxyl-terminal appended F5/8C module should increase the attachment of LamA to its natural substrates, for instance plant and fungal cell walls that often comprise cellulose, pectin, β-1,3-glucan, β-1,3-1,4-glucan, chitin and others. As a consequence, the F5/8C module increases the encountering frequency between the catalytic GH16 module and the β-1,3-glucosidic linkages within the complex substrates. In other words, the F5/8C module should be able to increase the "effective concentration" of the catalytic module on the surface of the substrates. The enhanced antifungal activity of the GH16 module by F5/8C on the growths of *C. albican *and *R. solani *[15], and *F. oxysporum *and *G. cingulata *(data presented here) support this notion.

A small fraction of the F5/8C module of LamA form dimers. The recombinant lactadherin C2 domain exists as a monomer in solution, but forms a dimer using a part of a β-strand (S7) as the contact area when it was packed in the crystal [33]. A similar result was observed in the crystal structure of the C2 domain of human factor V [34]. These observations suggest that dimerization under certain circumstances, such as high protein concentration, may be a general property of the DS domain. Dimerization of LamA, presumably anchored on cell wall by its S-layer homologous modules, may increase the binding valence of the protein and further increase the binding avidity of the *Paenibacillus *strain to its assimilated polysaccharides.

The mutations of W1679, W1688, and Y1768 did not cause dramatic changes in the binding affinity of the F5/8C module to chitin. These data suggest that substrate binding is not the primary function of these residues. Otherwise, a much greater impacts would have been expected. For example, mutations of the conserved tryptophan residues of the CBM on *Cellulomonas fimi *CenA caused 30- to 50-fold decreases in cellulose-binding affinity [35]. Furthermore, mutations of the CBM on *Thermotoga maritima *Lam16A caused 30- to 150-fold decreases in celluolose-binding [36].

**Figure 9:**
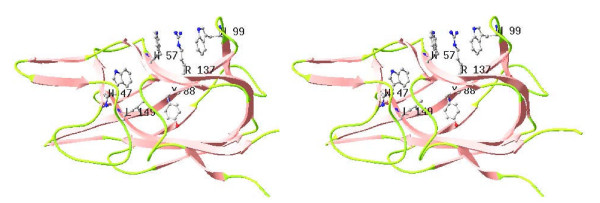
**Structure of the C2 domain of human factor V**. A ribbon plot is shown highlighting the β-sandwich fold. Side chains labeled W47, W57, Y88, W99, R137, L149 correspond to W2119, W2129, Y2160, W2171, R2209, and L2221 of mature factor V, respectively.

In the present study, the effect of mutation of the F5/8C module on protein expression and stability varied. Spatial locations of the mutated residues in the module are proposed in light of the crystal structure of the C2 domain of factor V (Fig. 9) to address the implications of these mutations. Conserved W1688, R1756, and W1729 (corresponding to W57, R137, and W99, respectively, in Fig. 9) are clustered on the surface such that the two indole groups sandwich the guanidinium group of R1756. The mutation W1729A results in the formation of inclusion bodies, which implies a critical role of the tryptophan in forming the β-sandwich fold. This suggestion is supported by the high mutational frequency of W163 in retinoschisin, the protein responsible for X-linked juvenile retinoschisis [37]. Mutation at W1688 also destabilized the protein, as demonstrated by a decrease in the Tm of 5.5 degrees. These results confirm the critical role of the cation-π interaction within the conserved triad in maintaining the structural integrity of DS domains [37,38]. W1679, Y1714, and Y1768 (corresponding to W47, Y88, and L149 respectively in Fig. 9) are buried within the protein. Replacement of Y1714 with alanine decreased the Tm from 57.8°C to 53.3°C. In addition, Y1714A had the most significant change in the CD spectrum. Together, these data indicate that the hydrophobic force involving Y1714 is important for the stability of the β-sandwich fold. In contrast, the structural roles of W1679 and Y1768 were less important based on the insignificant effects of these mutations on both the Tm values and the CD spectra.

## Conclusion

There is increasing documentation that bacterial glycoside hydrolases contain DS domains. However, the properties and functions of this domain are mostly speculative rather than characterized. Therefore, in this study, we characterized the DS domain in LamA of *Paenibacillus *sp. BCRC 17245. The DS domain can bind a variety of polysaccharides and maximize the inherent catalytic function of LamA. Furthermore, the importance of the conserved aromatic residues in the protein's conformation and stability can be interpreted based on the structure of mammalian DS domains, despite no more than 16% similarity in amino acid sequences between the domains of LamA and mammalian proteins. This suggests that the core of the β-sandwich fold has been conserved while the DS domain has evolved to have various ligand-binding functions. The W-R-W triad is particularly critical for the formation and maintenance of the β-sandwich fold based on the previous study on retinoschisin, crystal structure of mammalian DS domains, and data presented here.

## Competing interests

The authors declare that they have no competing interests.

## Authors' contributions

YMC carried out plasmid construction, protein purification, and biochemical analysis of the F5/8C module of LamA. FCH performed anti-fungal activity assay. MM participated in the design of the study and drafted the manuscript. All authors read and approved the final manuscript.
